# Case of Rupture of Aorta

**Published:** 1885-09

**Authors:** Henry Marshall

**Affiliations:** Clifton


### CASE OF RUPTURE OF AORTA. By Henry Marshall, M.D., F.R.S.E., Clifton.

E. S., æt. 35, housemaid in a private family, had been feeling vaguely ill and out of sorts (though able to do her work) for some two or three weeks before I saw her on January 24th, 1885. She was then in bed, on account of what appeared to be an ordinary catarrh, from which she had recently recovered in two or three days, but complained of great sleeplessness. For this I prescribed 10 grs. of hydrate of chloral and 15 grs. of bromide of potassium at bedtime, which she took for two nights, and when I saw her on the afternoon of the 31st she was downstairs, having resumed her work, and feeling, as she assured me, quite well again, if she could only sleep better at night: for although the first night on which she took the draught she had slept well, the next one she had been very restless. I prescribed 20 grs. of bromide of potassium with ♏xxx of aqua lauro cerasi, to be taken at bedtime. About 11 that night I received an urgent summons to see her at once, and as she lived very near my own house, I was in her room a few minutes afterwards, when I found her sitting on the side of her bed, supported by a fellow-servant, quite dead.

I received the following account: She had appeared during the day and evening in excellent spirits, remarking how much better she felt. After taking a hearty supper, she went upstairs to her bedroom. The nurse, who occupied a room next to hers, heard her cough a good deal while she was undressing; but shortly afterwards, when the cook, who slept in the same room, entered, she noticed that the housemaid appeared to be sleeping quietly, so she undressed and got into bed without speaking to her, and soon fell asleep, but was shortly after awakened by hearing her fellow-servant walking about the room and crying out that she was poisoned. She called to the nurse, who, having hot water ready, gave her some to drink, with the intention, which was partially successful, of making her sick; and at the same time there was a small relaxed action of the bowels. The breathing now became very laboured, and had ceased a minute or two before I entered the room. The interval between her getting out of bed and her ceasing to breathe was, as far as I could ascertain, about a quarter of an hour.

Being at the time unable to account for the cause of death, and thinking it possible that it might be the result of an overdose of aqua lauro cerasi, or some other poison,

I requested my friend Dr. Markham Skerritt to make a post-mortem examination, which he did on the day following her death; and I am indebted to him for the following account of the appearances he found:

"When the pericardium was opened, the aorta, from its origin for about an inch, was found to be adherent to the surrounding parts by recent lymph, its surface being roughened and reddened, and its texture softened; the vessel was here somewhat dilated. When the aorta was opened, it was found that the internal and middle coats were split across right round the circumference of the vessel, about $\frac{3}{4}$ inch above the aortic valves, and that from the site of rupture down to the valves these coats were separated from the external, so that the separated portion hung down free in the blood-current. On the distal side of the rent the separation of the coats extended for only about the sixteenth of an inch. The edges of the ruptured coats were as clean and sharp as if cut with a knife. There was a small amount of superficial atheroma in the vessel, but there was no evidence that the rupture had originated in an 'atheromatous ulcer.'"

I may observe, that my attendance on the case during the week previous to her death being of a casual nature, and there being no symptoms, unless perhaps the sleeplessness, suggesting any form of cardiac disease, I did not make any careful auscultation of the heart, although on the first day I saw her I made a general examination of the chest, and noticed no abnormal sounds; nor is it probable that any existed. The lesion, I imagine, took place only a quarter of an hour before her death; but if so, it is curious that it should have occurred when she was lying quiet and asleep.

